# Problem Adaptation Therapy for Pain (PATH-Pain): A Psychosocial Intervention for Older Adults with Chronic Pain and Negative Emotions in Primary Care

**DOI:** 10.3390/geriatrics2010005

**Published:** 2017-01-16

**Authors:** Dimitris N. Kiosses, Lisa D. Ravdin, Amy Stern, Ruth Bolier, Cara Kenien, M. Carrington Reid

**Affiliations:** 1 Weill Cornell Institute of Geriatric Psychiatry, White Plains, NY 10605, USA; dkiosses@med.cornell.edu (D.N.K.); afs9001@med.cornell.edu (A.S.); 2 Weill Cornell Medicine, 428 East 72nd Street, Suite 500, New York, NY 10021, USA; ldravdin@med.cornell.edu; 3 Academic Medical Center, University of Amsterdam, Meibergdreef 69, 1105 BK Amsterdam, The Netherlands; a.r.bolier@amc.uva.nl; 4 Weill Cornell Medicine, 525 East 68th Street, New York, NY 10065, USA; cak2017@med.cornell.edu

**Keywords:** elderly, chronic pain, negative emotions, cognitive impairment, behavioral therapy

## Abstract

Chronic pain is highly prevalent in older adults, contributes to activity restriction and social isolation, disrupts family and interpersonal relationships, and poses a significant economic burden to society. Negative emotions such as sadness, anxiety, helplessness, and hopelessness are associated with chronic pain and contribute to poor quality of life, impaired interpersonal and social functioning, and increased disability. Psychosocial interventions for older adults with chronic pain have been historically developed for, and are almost exclusively delivered to, cognitively intact patients. Therefore, many older adults with chronic pain and comorbid cognitive deficits have limited treatment options. Our multidisciplinary team developed Problem Adaptation Therapy for Pain in Primary Care (PATH-Pain), a psychosocial intervention for older adults with chronic pain, negative emotions, and a wide range of cognitive functioning, including mild-to-moderate cognitive impairment. In the current article, we describe the principles underlying PATH-Pain, review the steps taken to adapt the original PATH protocol, outline the treatment process, and present a case illustrating its potential value.

## 1. Introduction

Chronic pain is highly prevalent in older adults and associated with emotional disturbances, activity restriction, social isolation, falls, and sleep impairment [1]. Its negative effects extend beyond the individual to disrupt both family and social relationships [2,3]. Chronic pain also poses a significant economic burden on society. The annual cost of pain in the United States is nearly 30% higher than the cost of cancer and diabetes combined [4]. As the population ages, prevalence rates for pain are expected to increase, thereby increasing its public health impact.

Negative emotions, including sadness, anxiety, helplessness, and hopelessness, are associated with chronic pain [5,6,7] and contribute to poor quality of life, impaired interpersonal and social functioning, and increased disability [5,6,8]. The relationship of emotions with pain intensity and pain-related disability is reciprocal. Specifically, (a) increased negative emotions and decreased positive emotions modulate or alter pain perception, contributing to pain-related disability [9]; and (b) pain contributes to increased negative emotions, decreased positive emotions, and consequent disability [10]. A psychosocial intervention focusing on reducing negative emotions and increasing positive emotions may consequently reduce pain intensity and pain-related disability.

Existing psychosocial interventions for older adults with chronic pain have been developed for, and are almost exclusively delivered to, cognitively intact patients [11]. Therefore, many older adults with chronic pain and cognitive deficits have limited treatment options. This is a significant limitation in clinical care because cognitive impairment is highly prevalent in patients with chronic pain and contributes to detrimental consequences, including undertreatment, low adherence to treatment, disability, and poor quality of life for patients and their families [12,13]. To the best of our knowledge, no behavioral intervention has been specifically developed to address pain in older adults with co-occurring cognitive impairment and negative emotions.

We developed Problem Adaptation Therapy for Pain in Primary Care (PATH-Pain), which targets older adults with comorbid pain, negative emotions, and a wide range of cognitive functioning, including cognitively intact patients as well as those with mild-to-moderate cognitive impairment. Problem Adaptation Therapy for Pain in Primary Care (PATH-Pain) is an adapted version of Problem Adaptation Therapy (PATH). PATH specifically seeks to reduce depression and improve functioning in older adults with comorbid depression and cognitive impairment. In a randomized controlled trial, participants randomized to receive PATH (vs. supportive) therapy demonstrated significant reductions in depression and disability [14,15]. As most older adults with chronic pain are treated in primary care, [16] PATH-Pain was specifically developed for use by older patients in the primary care setting to increase both its reach and impact.

In the current article, we describe the principles underlying PATH-Pain, review steps taken to adapt the original PATH protocol, outline the treatment process, present a case illustrating the potential value of the intervention, and highlight potential challenges to its implementation.

## 2. Methods

### 2.1. Principles of PATH-Pain

**Figure 1:**
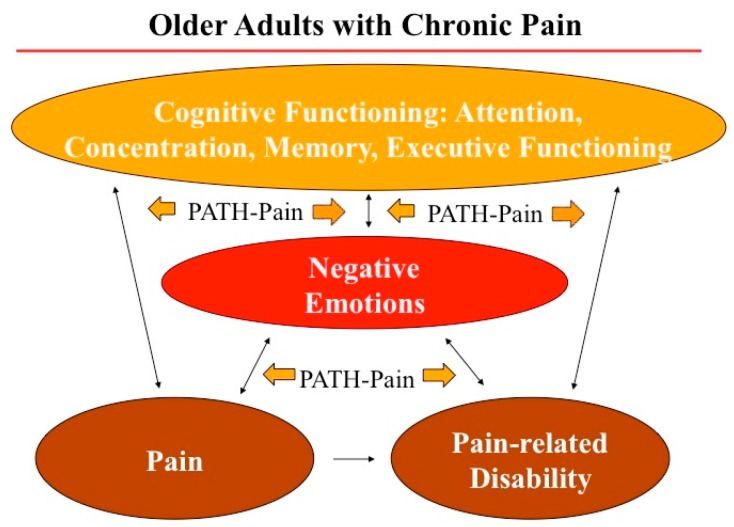
PATH-Pain Effects on Negative Emotions, Pain and Pain-Related Disability.

PATH-Pain is a psychosocial intervention for older adults with chronic pain, negative emotions, and a wide range of cognitive functioning, including mild-to-moderate cognitive impairment, delivered by a mental health professional (e.g., social workers or psychiatric nurses) in primary care. PATH-Pain aims to (a) reduce negative emotions (e.g., sadness, irritability, anger, hopelessness, helplessness, guilt, anxiety); (b) promote positive emotions (e.g., pleasure); (c) break the vicious cycle of inactivity that is promoted by negative emotions, pain, and physical limitations; (d) improve communication between the patient and primary care physician; and (e) address barriers to pain and psychological treatment (Figure 1). PATH-Pain utilizes a structured yet personalized approach to help patients address these difficulties.

PATH-Pain employs the following tools to achieve its goals: (a) education of patients on pain, pain treatment, and the effect of emotions on pain and pain-related disability; (b) identification of triggers of negative emotions and pain; (c) emotion regulation techniques (Table 1) to reduce negative emotions and increase positive emotions related to pain and pain-related disability, such as situation selection (e.g., select the situations the patient is exposed to), situation modification (e.g., modify the situation the patient is exposed to), attentional deployment (e.g., shift patient’s attention within a situation), cognitive change (e.g., change how the patient thinks about the situation), and response modulation (e.g., use direct efforts to alter patient’s emotional responses) [17]; (d) a simplified problem-solving approach to break the vicious cycle of inactivity, help patients engage in pleasurable or rewarding activities, and improve patient-physician communication; (e) attention techniques to reduce pain intensity; (f) environmental adaptations (e.g., notes, calendars, signs, and step-by-step activity plans) to bypass cognitive and functional limitations; and (g) selective engagement a willing and available significant other (e.g., family member, friend, or professional caregiver). Using these tools, PATH-Pain may be able to decrease everyday stressors, empower patients, instill hope, reduce helplessness, and reduce pain perceptions and pain-related disability [18]. PATH-Pain is delivered in primary care and its hands on techniques can be easily used by cognitively intact patients. Furthermore, unlike existing psychosocial treatments for older adults with chronic pain, PATH-Pain may be helpful for cognitively impaired older adults by employing environmental adaptation tools and by selectively incorporating caregiver participation in treatment (Table 2).

### 2.2. Adapting the Original PATH Protocol

A multidisciplinary panel, consisting of experts in geropsychology and psychosocial interventions (DK, who is the PATH developer), neuropsychology (LDR), social work (AS), and geriatric and pain medicine (CR), oversaw the adaptation, and finalized the PATH-Pain manual. To identify the unmet needs of older adults with chronic pain, negative emotions, and a wide range of cognitive functioning, and to guide the adaptation of the PATH protocol and create PATH-Pain, we conducted an extensive review and analysis of the literature and evaluated psychosocial treatments designed for older adults with comorbid pain and emotional disorders. Following this review and analysis, panel members conducted (1) in-depth clinical interviews with 10 older adults with chronic pain, negative emotions, and a wide range of cognitive functioning to gain insight into the respective roles of negative and positive emotions in the target population; and (2) a pilot study of 102 older adults with chronic pain in primary care to identify the types of problems these patients routinely face, examine the effects of negative and positive emotions on pain and pain-related disability, and assess the vicious cycle of inactivity by describing the pleasurable activities older adults typically pursue and the extent to which these activities are modified (or eliminated altogether) because of pain (PI: Reid) [19]. Data collected from these activities informed the adaptation of the PATH treatment manual for use in older adults with comorbid pain, negative emotions, and cognitive impairment. We are also currently conducting a small-scale clinical trial of PATH-Pain in primary care (MPI’s: Kiosses, Reid). Data from this initiative will be used to further adapt the protocol, as appropriate.

### 2.3. PATH-Pain Treatment Phases

PATH-Pain has 4 main treatment phases delivered weekly over the first 8 weeks then monthly thereafter over the next 4 months:

*1*. Initial Assessment and Treatment Planning (Weeks 1 & 2)
*2*. Treatment Implementation (Weeks 3 to 7)
*3*. Treatment Termination (Week 8)
*4*. Treatment Booster Sessions (Delivered once a month in weeks 12, 16, 20, and 24)

#### 2.3.1. Initial Assessment and Treatment Planning

During Sessions 1 and 2, the PATH-Pain therapist collects information about the patient’s pain, pain-related disability, and pain treatments; ascertains the patient’s physical and cognitive strengths and limitations; evaluates the significant other’s ability and availability to assist in the therapy; and helps patients identify triggers of negative emotions (i.e., problems, situations, or concerns that contribute to negative emotions such as depressed mood, irritability, anger, anxiety, hopelessness, helplessness, and worthlessness) as well as triggers of positive emotions (e.g., pleasurable or rewarding activities). During this phase, the patient receives a PATH-Pain Handbook that describes PATH-Pain and summarizes the individual techniques.

In this phase, the therapist analyzes triggers (problems/situations/concerns) of negative emotions and creates a treatment plan to reduce them. At the same time, the therapist creates a list of potential pleasurable and rewarding activities to increase pleasure and motivation. The therapist and patient identify barriers to engagement in pleasurable activities and devise a step-by-step plan to overcome them.

Depending on the patient’s cognitive limitations, the treatment plan may include PATH-Pain environmental adaptation tools (e.g., reminders and notes) and the involvement of a willing and available significant other in the treatment process. PATH-Pain utilizes planning tools (e.g., a step-by-step division of tasks), attentional tools (e.g., minimization of environmental distractions) visual tools (e.g., calendars and note pads), and acoustic tools (e.g., times and alarms). These tools do not improve the patient’s cognitive impairment but reduce its functional consequences by providing compensatory mechanisms, which in turn can reduce the patient’s emotional response. A willing and available significant other (e.g., spouse) may selectively participate in aspects of PATH-Pain treatment, helping to articulate triggers, executing plans to address them, initiating pleasurable activities, selecting and implementing PATH-Pain tools, and providing valuable insight and feedback on the treatment process. These tools and the involvement of a significant other were very effective in helping to reduce depression and disability levels among older adults with depression and cognitive impairment in the original PATH intervention [18].

Triggers of negative emotions vary among individuals and include problems and situations such as tension in interpersonal relationships, frustration with pain or pain treatments, miscommunication with the primary care physician, lack of engagement in activities, and functional impairment. A recent analysis of triggers of negative emotions in older adults with chronic pain revealed that at least half of the problems were not directly associated with pain but they were still extremely bothersome to patients [19]. Reducing the negative emotions associated with these problems may provide relief by improving tolerance to pain and reducing pain-related disability.

The therapist contacts the patient’s primary care physician (PCP) at the start of treatment to provide information about the intervention and solicit pertinent information about the patient’s medical condition and current treatments for pain. The therapist specifically asks the PCP to comment on problems the patient has that possibly impact his or her mood, pain, and/or functioning, as well as any negative emotions (such as depression, anxiety, helplessness, and hopelessness) that have interfered with pain treatments in the past. The therapist maintains contact with the patient’s PCP throughout the treatment.

#### 2.3.2. Treatment Implementation

During Sessions 3–7, the therapist reviews the principles of PATH-Pain, selects a particular trigger of negative emotions, identifies ways to reduce negative and increase positive emotions, facilitates the implementation of the treatment plan with the patient (and significant other when appropriate), evaluates the effectiveness of the treatment plan, and revises it accordingly. During this phase, the therapist enhances communication between the therapist, patient, and the PCP, identifies and corrects any misunderstandings, and modifies expectations to pain treatment as appropriate. For example, some patients have unrealistic treatment expectations (i.e., “I expect the pain treatment to make my pain go away entirely”) that may not be met. Finally, the therapist identifies how negative emotions interfere with the PATH-Pain treatment and utilizes emotion regulation techniques to reduce these negative emotions (such as helplessness, helplessness, and irritability), promote effective communication between the therapist and patient, and prevent discontinuation of treatment.

#### 2.3.3. Treatment Termination

During this session, the therapist discusses the pros and cons of the PATH-Pain treatment, highlights the most successful techniques that were successful in reducing negative emotions, promoting positive emotions, and reducing pain intensity and pain-related disability, and creates a plan as to how the patient will utilize these techniques in the future. The therapist provides the patient with a written personalized summary of treatment that describes the most important aspects of treatment.

#### 2.3.4. Monthly Booster Sessions

During these sessions, the therapist assesses the emotional state of the patient, reviews the PATH-Pain strategies, evaluates their effectiveness in maintaining treatment gains, modifies these strategies as needed, and identifies and addresses any new problems the patient faces. Finally, the therapist explores any pain treatment issues and communicates any concerns to the PCP.

## 3. Case Presentation

Ruby is an 82 year-old woman with mild cognitive deficits who resides in an apartment with her husband Mort, who is 90-years old and has mild dementia. Ruby suffers from severe back and knee pain due to spinal stenosis and arthritis. Both conditions have been present for more than 20 years. Medications used to treat her pain include scheduled methadone and venlafaxine, as well as prn use of oxycodone for breakthrough pain. Both the PCP and patient agree that this treatment regimen has not measurably reduced her pain. She continues to experience pain scores of 7 or greater (on a scale of 0–10) when walking, bending her back, and turning over or moving her lower extremities in bed. Her pain is less severe when she sits in a chair. At the initial visit, she is alert and fully oriented, expresses thoughts in an organized and coherent manner, and endorses mild short-term memory difficulties. Ruby and Mort recently hired a home health aide to help with shopping, food preparation, housekeeping, and laundry tasks. Because of Mort’s cognitive impairment, Ruby declined to involve him in the treatment.

*Negative Emotions, Pain, Pain Treatment, and Inactivity*: The negative emotions associated with Ruby’s pain included sadness, disappointment, anger, helplessness, and hopelessness. Specifically, Ruby expressed feelings of being let down by the medical system and disappointed by the many previous unsuccessful attempts to manage her pain. As a result, Ruby voiced skepticism about whether PATH-Pain could help her and began each session by expressing her inclination to discontinue treatment. To reduce these negative emotions and prevent them from interfering with treatment, the therapist was empathic to the patient’s concerns, explained how these emotions negatively affect her everyday life and her pain treatment, and highlighted that reducing these negative emotions may improve Ruby’s quality of life. With the patient’s help, the therapist devised a plan to reduce these negative feelings.

During the weekly treatment sessions, the following techniques were implemented to reduce Ruby’s negative emotions. The emotion regulation strategy of *attentional deployment* was particularly useful; Ruby identified several pleasurable and rewarding activities that could shift her attention away from her negative emotions and experience of pain. The list of activities included those she could still do, as well as activities that she no longer did because of her pain. With the help of the therapist, Ruby wrote out a plan indicating that she would initiate a particular activity such as “Go sit at the kitchen table and start a crossword puzzle” when she found herself focused on her pain or her sadness, anger, or helplessness. Ruby stated that distraction was successful in reducing her negative emotions and her perception of pain. In addition, engaging in pleasurable activities helped her to break the cycle of inactivity fueled by pain and negative emotions.

The strategy of *cognitive change*, i.e., changing perspective to reduce negative emotions, was also successfully employed. Ruby was discouraged, angry, and disappointed because, even though there were times that the pain was reduced, the pain still came back afterwards. The therapist encouraged Ruby to challenge her thinking by looking at the situation from a different perspective. Statements such as “a break from thinking about my pain, even a short one, is worthwhile even if the pain does not go away completely” were written down to be referred to in times of need.

*Financial Stressors and Memory Difficulties:* Ruby and the therapist identified two additional areas of concern: financial stressors and short-term memory problems. During the course of her treatment, Ruby, with the help of her therapist, created a plan to reduce expenses and used the emotion regulation strategies of attentional deployment and situation modification to reduce the feelings of anxiety and helplessness associated with her financial stress. The strategy of cognitive change was used to reduce anger and frustration with short-term memory problems. The PATH-Pain therapist reinforced Ruby’s use of compensatory tools for her memory difficulties, such as writing all appointments in her calendar, making lists, and leaving notes for herself as reminders.

*Reduction of Pain Intensity:* Ruby noticed that she focused less on her pain when visiting with friends than when she was alone. She had a few friends who lived in her building that would at times drop-in and join her for coffee, but Ruby did not typically initiate social engagements. By contacting friends and scheduling dates, Ruby used *situation selection* to trigger positive emotions and lessen her experience of pain.

The therapist also educated Ruby about deep breathing and progressive relaxation exercises as a means to reduce negative emotions and her perception of pain. The therapist provided print-outs of progressive relaxation scripts and conducted a breathing exercise in session; however, Ruby reported that this *response modulation* strategy was not particularly helpful.

*Communication with PCP:* Despite Ruby’s frustration with the medical establishment, she continued to investigate new medical options to treat her pain. Ruby considered a local acupuncturist but had difficulty asking the PCP for the referral. With the help of the therapist, Ruby devised a plan to help her request the referral from her PCP. Ruby was encouraged to see that her PCP supported this pursuit, which reduced her feelings of helplessness and hopelessness. The therapist communicated with Ruby’s PCP at various points during the treatment to receive updates on her medical status and to report on useful emotion regulation strategies.

At the conclusion of the weekly sessions, Ruby was given a personalized treatment summary outlining the problems and pleasurable activities targeted in treatment, the related emotions, and the methods used to address them. Ruby was advised to reference the summary to help her manage emotions in the future. A copy of the summary was also given to her PCP so that he could reinforce and encourage continued use of the techniques Ruby learned during the PATH-Pain therapy.

As a result of incorporating PATH-Pain, Ruby was able to successfully reduce her negative emotions and promote positive emotions, thereby improving her perception of the impact of pain as well as the degree to which pain limited her activity.

## 4. Potential Challenges Implementing PATH-Pain

While we were able to successfully implement the protocol in the above case and achieve positive results, we recognize there are potential barriers to implementing a psychosocial intervention for older patients with negative emotions and chronic pain in primary care. The presence of significant pain will serve as a barrier to traveling to the doctor’s office to undergo the therapy for some older patients, while inclement weather conditions will make travel difficult for others. Co-morbid medical issues (e.g., visual impairment and gait disorders) and competing demands (e.g., doctors’ appointments, physical therapy sessions, and serving in a caregiver role) can also interfere with scheduling the weekly therapy sessions. Therefore, PATH-Pain therapists must be flexible regarding the dates and times of day that sessions are scheduled. The therapist should be responsive to patients’ requests to reschedule appointments, if needed, to accommodate these concerns. By housing the intervention in the primary care setting, however, we increase the likelihood that the route of transportation is reasonable, as this is a location that patients have chosen for their routine medical care.

Some older patients with chronic pain may have doubts that a psychosocial intervention will improve their quality of life. For these patients, the PATH-Pain therapist reinforces the nature of the intervention and instills a sense of hope and open-mindedness regarding the possible benefits of this treatment approach. Individuals who have never participated in psychotherapy will require further orientation to the process. In addition, the success of PATH-Pain will likely be impacted by the degree of integration that can be achieved in the primary care setting. The PATH-Pain therapist will determine the most effective manner of communication with each patient’s PCP at the start of treatment, e.g., e-mail, electronic medical record, phone, or in-person. PCPs have large patient caseloads and time constraints, so establishing an on-going dialogue about each patient’s therapy will be important to do. PATH-Pain therapists will inquire about patients’ past experiences with behavioral therapy (if any) and any barriers the PCP feels their patients might encounter undergoing PATH-Pain therapy, thereby leveraging the physician’s knowledge of their patients.

## 5. Conclusions

Chronic pain is highly prevalent in older adults and associated with multiple comorbidities as well as functional compromise. Chronic pain is often accompanied by negative emotions, which can further exacerbate the perception of pain as well as negatively impact functional independence. Given that many older adults also experience cognitive changes, psychosocial interventions for older adults must factor in cognitive compromise if the intervention is to be effective. PATH-Pain is a promising psychosocial intervention that addresses chronic pain by providing techniques that help affected patients to decrease the impact of negative emotions, increase the experience of positive emotions, and use compensatory mechanisms for cognitive deficits to promote application of the strategies in a manner that is tailored for each patient’s specific situation. Importantly, this therapy can be applied in primary care settings where it can reach many potential candidates for treatment.

## Figures and Tables

**Table 1:** PATH-Pain Emotion Regulation Strategies.

Ways to Regulate Emotions	Goal	PATH-Pain
Situation Selection	Select situations patient is exposed to	Identify situations that trigger negative emotions (e.g., sadness, irritability, anger, helplessness, hopelessness, worthlessness) and pain. Devise a plan to avoid these situations or reduce their frequency. Caregivers/significant others may contribute by reinforcing use of the plan, identifying barriers to the plan, and encouraging patient to stay on course.
		Identify situations and activities that trigger positive emotions. Devise a plan to promote activities.
Situation Modification	Change situation patient is exposed to	Devise a plan to modify situations that trigger negative emotions and pain. Modifications include environmental changes, engagement in activities (i.e., have a list of pleasurable or rewarding activities), or other techniques (i.e., change the focus on something positive, such as calling a friend).
		Caregiver/significant other may help in the implementation of the environmental changes, the engagement of pleasurable and rewarding activities, and the employment of the techniques.
Attentional Deployment	Shift patient’s attention within a situation	Attention, planning, and visual and acoustic tools (e.g., notes, shaping procedure to sustain attention, step-by-step plan, timers) are used to shift attention once the pain and negative emotions occur. Caregiver/significant other may help identify barriers to the implementation of the techniques, offer suggestions for possible solutions, and discuss them with the patient and the therapist.
Cognitive Change	Change how situation is perceived	Cognitive reappraisal is used to change the patient’s perspective to reduce negative emotions, improve positive emotions, and reduce pain. Patients, with therapist’s help, change their perspective to reduce negative emotions, increase positive emotions, and develop a realistically hopeful approach to learn to live with pain. Caregiver/significant other may reinforce the cognitive reappraisal techniques as well as identify barriers to their implementation.
Response Modulation	Direct efforts to alter emotional responses	Promote utilization tools during or after the experience of negative emotions or pain. Therapist utilizes techniques to help reduce potential tension between patient and caregiver. Caregiver/significant other may participate in the implementation of these strategies, identify potential barriers to their implementation, and participate in the identification of solutions to these barriers.

**Table 2:** Main Elements of PATH-Pain.

Is appropriate for use by patients with cognitive impairment
Encourages caregiver participation
Uses environmental adaptation tools to bypass cognitive and functional limitations (e.g., signs, calendar, and notes)
Is developed for use in primary care
Explicitly promotes communication with primary care provider
General Psychotherapeutic Techniques
Employs activity scheduling
Employs problem solving
Emotion Regulation Strategies
Employs situation selection
Employs situation modification
Employs attentional deployment
Employs cognitive reappraisal
Employs response modulation
